# Analysis of long intergenic non-coding RNAs transcriptomic profiling in skeletal muscle growth during porcine embryonic development

**DOI:** 10.1038/s41598-021-94014-w

**Published:** 2021-07-27

**Authors:** Wenjuan Zhao, Zijing Li, Quan Liu, Su Xie, Mengxun Li, Yuan Wang, Changchun Li

**Affiliations:** 1 Key Laboratory of Agricultural Animal Genetics, Breeding, and Reproduction of the Ministry of Education and Key Laboratory of Swine Genetics and Breeding of the Ministry of Agriculture, Huazhong Agricultural University, Wuhan, 430070 China; 2 Guangxi Yangxiang Co., Ltd. Production Center, Guigang, 537131 China

**Keywords:** Biological techniques, Genetics, Molecular biology

## Abstract

Skeletal muscle growth plays a critical role during porcine muscle development stages. Genome-wide transcriptome analysis reveals that long intergenic non-coding RNAs (lincRNAs) are implicated as crucial regulator involving in epigenetic regulation. However, comprehensive analysis of lincRNAs in embryonic muscle development stages remain still elusive. Here, we investigated the transcriptome profiles of Duroc embryonic muscle tissues from days 33, 65, and 90 of gestation using RNA-seq, and 228 putative lincRNAs were identified. Moreover, these lincRNAs exhibit the characteristics of shorter transcripts length, longer exons, less exon numbers and lower expression level compared with protein-coding transcripts. Expression profile analysis showed that a total of 120 lincRNAs and 2638 mRNAs were differentially expressed. In addition, we also performed quantitative trait locus (QTL) mapping analysis for differentially expressed lincRNAs (DE lincRNAs), 113 of 120 DE lincRNAs were localized on 2200 QTLs, we observed many QTLs involved in growth and meat quality traits. Furthermore, we predicted potential target genes of DE lincRNAs in cis or trans regulation. Gene ontology and pathway analysis reveals that potential targets of DE lincRNAs mostly were enriched in the processes and pathways related to tissue development, MAPK signaling pathway, Wnt signaling pathway, TGF-beta signaling pathway and insulin signaling pathway, which involved in skeletal muscle physiological functions. Based on cluster analysis, co-expression network analysis of DE lincRNAs and their potential target genes indicated that DE lincRNAs highly regulated protein-coding genes associated with skeletal muscle development. In this study, many of the DE lincRNAs may play essential roles in pig muscle growth and muscle mass. Our study provides crucial information for further exploring the molecular mechanisms of lincRNAs during skeletal muscle development.

## Introduction

Skeletal muscle is an important component of the body in mammals, mainly involved in the growth and development of the body. Skeletal muscle abnormalities can lead to physical dysfunction such as Muscular dystrophy, idiopathic inflammatory myopathies and cardiomyopathy^1–4^. During the past decades of molecular biology study, great progress has been made on the molecular mechanism underlying the growth and development of porcine skeletal muscle^5^, for example, MyoD, MyF5 and MRF4 are involved in myogenesis and differentiation^6–8^. Additionally, studies have found that insulin-like growth factors (IGF1) can act as an activator of MAPK/ERK and PI3K/Akt signaling pathways to promote the proliferation and differentiation of muscle cells^9^, and IGF1 mediated pathway that the IGF1–Akt–mTOR pathway has been found to participate in positive regulation of muscle growth^10,11^. In recent years, the emergence of long intergenic non-coding RNA has become a new research hotspot in the molecular biological field, which provides a new way to advance the research on the mechanism of skeletal muscle development.

Long intergenic non-coding RNAs, which are a new class of RNA molecules longer than 200 nucleotides with little or no protein-coding capacity^12^. Recent evidences have established that lincRNAs have a significant role in regulating gene expression at epigenetic, transcriptional and post transcriptional levels^13,14^, they can perform essential functions during basic biological processes, such as chromatin modification^15^, imprinting^16,17^ , maintenance of pluripotency^18^. With the emergence and widespread application of high-throughput sequencing technology, thousands of lincRNAs have been identified in genome-wide analysis, more and more lincRNAs have been functionally validated. A recent study indicated that lincRNA-p21 is involved in regulating the proliferation and apoptosis of vascular smooth muscle cells by enhancing the activity of P53, providing a new target for the treatment of atherosclerosis^19^. Currently, studies on lincRNAs in porcine embryo development are less well understood, therefore, our analysis in the differences of lincRNAs at embryonic development stages will provide a good model for studying the mechanisms that regulate skeletal muscle development.

In the present study, we applied RNA sequencing to characterize global gene expression patterns of muscle tissues from Duroc on days 33, 65, and 90 and systematically analyzed the muscle expression profile during porcine skeletal muscle development^20^. We identified 228 putative lincRNAs and found that many lincRNAs differentially expressed. Moreover, we predicted the potential target genes of DE lincRNAs by cis or trans ways. Gene Ontology and pathways enrichment analysis showed that lincRNAs potentially regulated the process of protein-coding genes. An interactive network was performed to elucidate the interplay between DE lincRNAs and their potential target genes. This study of skeletal muscle of transcriptome profiles will provide a useful resource to further explore the understanding of mechanisms, besides, elucidating the underlying mechanisms of skeletal muscle growth and development will be helpful for the improvement of production benefits of pig.

### Summary of RNA-seq data mapping and transcripts assembly in Duroc

**Figure 1:**
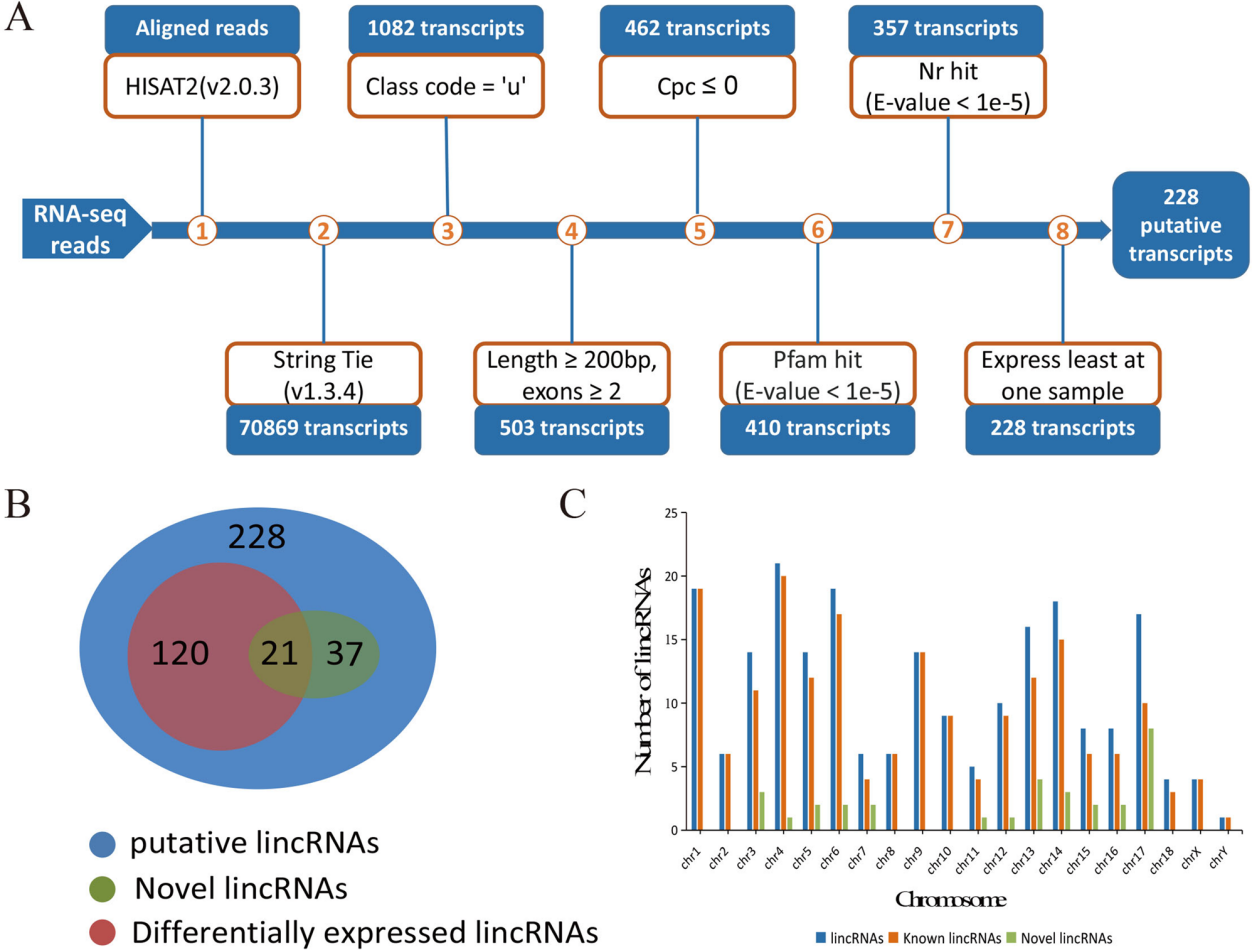
Overview of the identification pipeline for lincRNAs. **(A)** The identification pipeline of putative lincRNAs; **(B)** Venn diagram of putative lincRNAs, novel lincRNAs and differentially expressed lincRNAs; **(C)** The column diagram of chromosome distribution of putative lincRNAs.

In this study, we downloaded 9 RNA-seq libraries which contained 647,779,568 paired-end reads from the NCBI during three embryonic muscle developmental stages of Duroc. Samples were named separately 33d_1, 33d_2, 33d_3, 65d-1, 65d_2, 65d_3, 90d-1, 90d_2, 90d_3. After trimming and filtering, a total 636,985,862 clean reads were mapped to the annotated Sscrofa11.1 genome using HISAT2, we founded that approximately 95% of the quality-filtered reads were mapped and over 70% of the reads could be uniquely mapped to the genome (Table 1). Based on the result, StringTie was used to reconstruct the transcripts and merge into a file that obtained 70,869 transcripts. The RNA-seq process for identifying lincRNAs was shown in Fig. 1. Finally, 228 putative lincRNAs were identified, there were 191 lincRNAs that have been annotated in the pig reference genome database, and these known lincRNAs were distributed throughout all chromosomes. The remaining 37 lincRNAs have no overlap with the pig annotation database, they were separately distributed on chromosomes 3 to 8 and 11 to 18, and chromosome 17 was found to have the highest novel lincRNAs density.

**Table1:** Summary of data from RNA-seq.

Sample	Accession number	Raw reads	Clean reads	Mapped reads	Mapping ratio (%)	Uniquely mapping ratio (%)
33d_1	SRR9829616	74,174,368	72,887,888	54,060,532	95.43	74.14
33d_2	SRR9829617	65,334,814	64,482,010	45,701,718	94.90	70.88
33d_3	SRR9829614	77,428,960	76,451,044	58,773,616	95.96	76.88
65d_1	SRR9829615	73,747,044	71,946,704	52,038,372	95.09	72.33
65d_2	SRR9829612	71,704,676	70,420,214	50,268,952	94.95	71.38
65d_3	SRR9829613	69,401,348	68,207,232	48,032,548	94.76	70.42
90d_1	SRR9829610	65,996,802	65,243,880	47,417,538	95.18	72.68
90d_2	SRR9829611	71,715,594	70,405,728	50,090,100	95.00	71.14
90d_3	SRR9829609	78,275,962	76,941,162	54,391,950	94.87	70.69

### Characteristics analysis of identified lincRNAs

**Figure 2:**
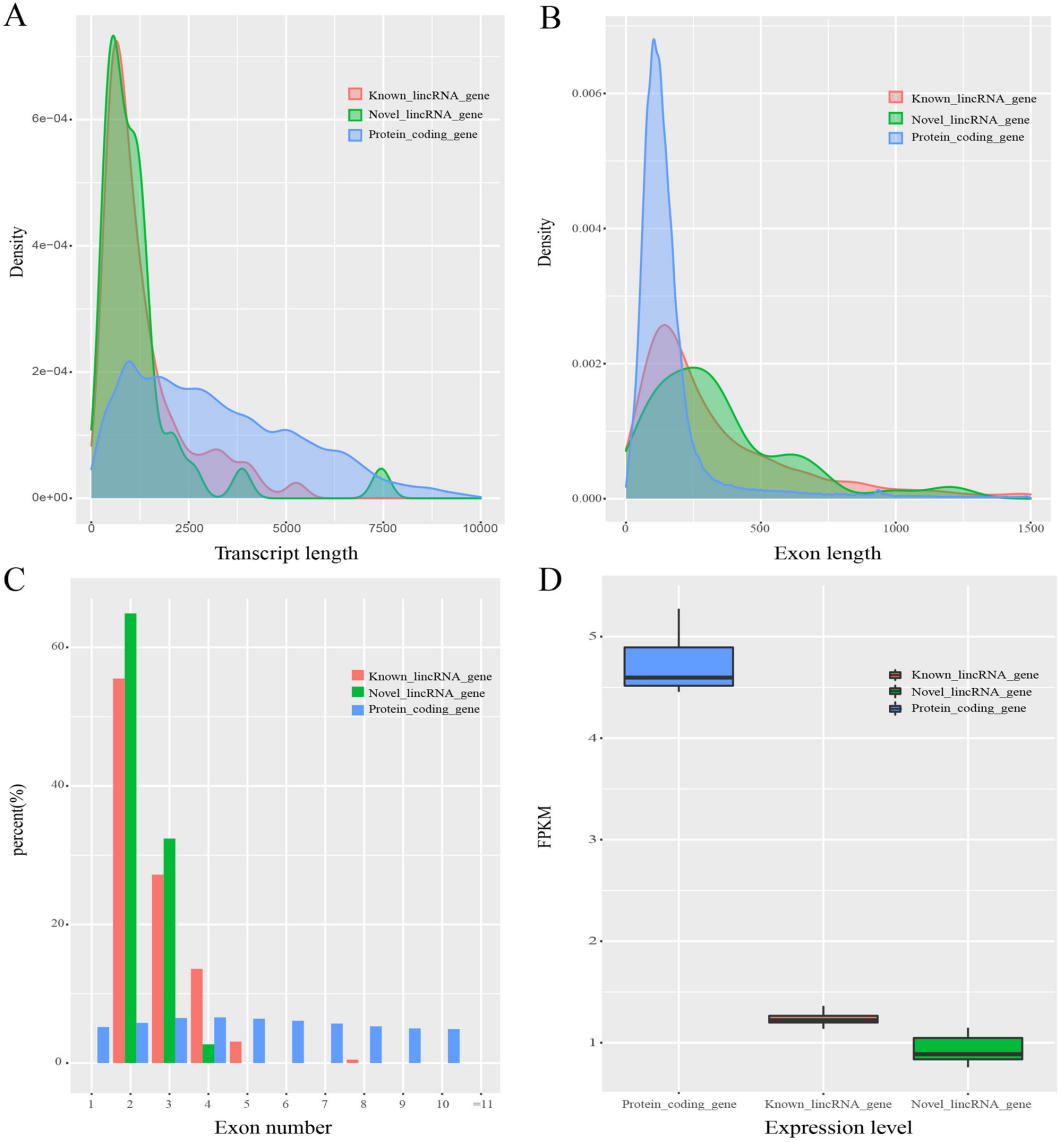
Characterization of lincRNAs compared with protein-coding genes; **(A)** Comparison of transcript length; **(B)** Comparison of exon length; **(C)** Comparison of numbers of exon; **(D)** Comparison of expression level.

Previous study showed that the difference of lncRNAs with protein-coding genes in pig^21^. However, sequence characteristic of lincRNAs during embryonic muscle development remains unclear. Based on the annotated information for the pig reference genome, we examined the characteristic of putative lincRNAs in transcript length, exon length, exon numbers and expression level compared with protein-coding genes. As a result, we observed that the average transcript length of known lincRNAs, novel lincRNAs and protein-coding genes were about 1377 bp, 1203 bp and 3296 bp, respectively. It followed that novel lincRNAs were similar to known lincRNAs and shorter than protein-coding genes in transcript length (Fig. 2A). In addition, the average exon length of known lincRNAs, novel lincRNAs and protein-coding genes were 515 bp, 505 bp and 284 bp, respectively. Although the average transcript length of lincRNAs was shorter, the average exon length of lincRNAs was longer than that of protein-coding genes (Fig. 2B). In exon numbers, our result showed that the exon numbers of lincRNAs were gathered at 2–5, while the average exon numbers of protein-coding genes were 11.6, we noticed that this result was consistent with the above two research (Fig. 2C). In normalized read counts expression level (FPKM), the average value of known lincRNAs, novel lincRNAs and protein-coding genes were 1.2, 0.9 and 4.7, respectively. We concluded that lincRNAs had a lower expression level compared with protein-coding genes. In general, lincRNAs were shorter in transcript length, but longer in exon length, had fewer exon, and were expressed at lower level compared with protein-coding genes (Fig. 2D). Which were highly consistent with previous reports^22,23^.

### Differential expression analysis of lincRNAs

**Figure 3:**
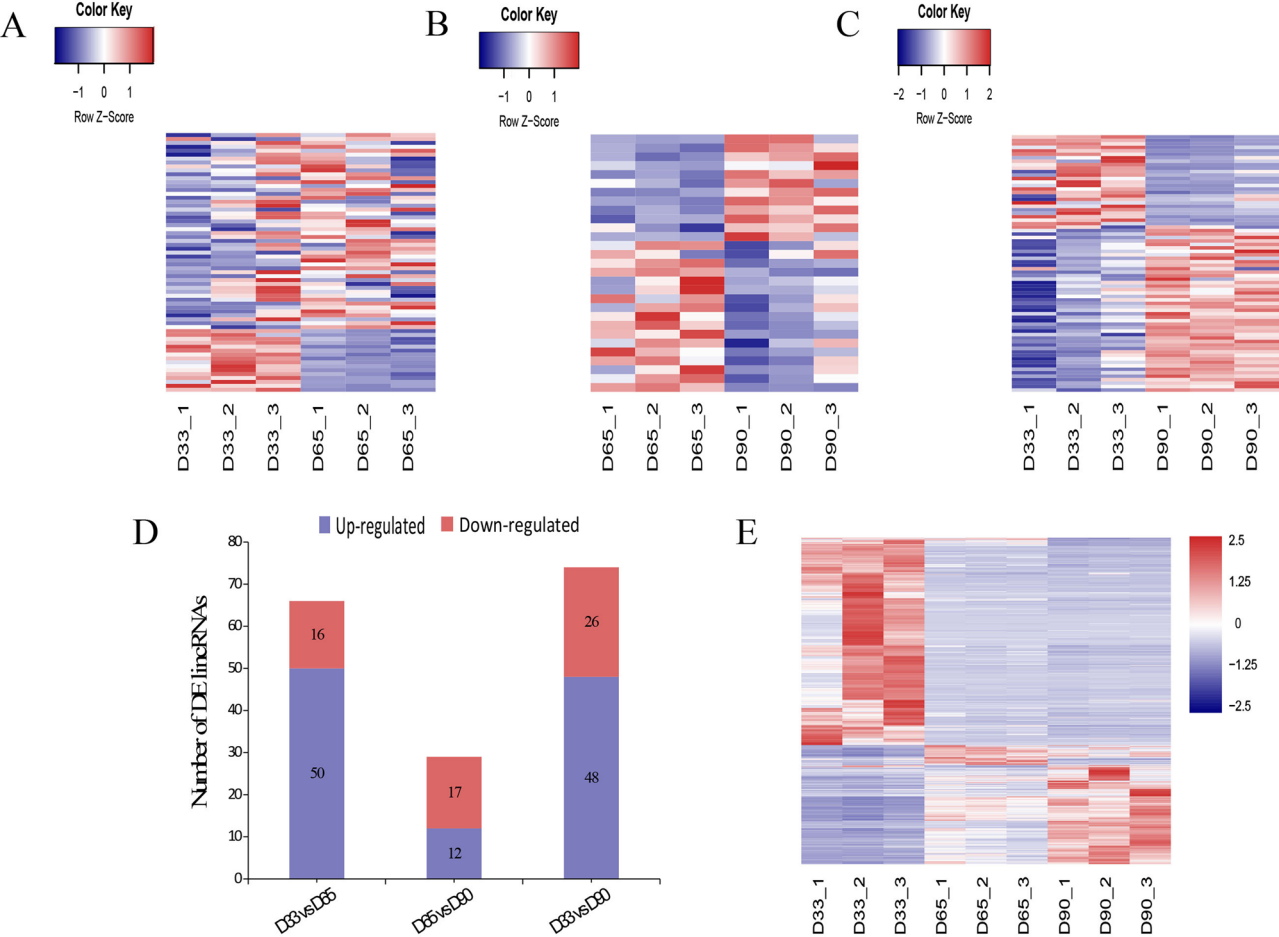
Heat map of differential expression analysis of lincRNAs and protein-coding genes during three developmental stages. **(A)** Heat maps of differentially expressed lincRNAs in D33 vs. D65 group; **(B)** Heat maps of differentially expressed lincRNAs in D65 vs. D90 group; **(C)** Heat maps of differentially expressed lincRNAs in D33 vs. D90 group; **(D)** Histogram of differentially expressed lincRNAs among the three groups; **(E)** Heat maps of differentially expressed protein-coding genes among the three groups.

To evaluate the differences in gene expression patterns during three developmental stages, DEseq2 was used to identify differentially expressed lincRNAs and protein-coding genes between two paired samples (D33 vs. D65; D65 vs. D90; D33 vs. D90). when |fold change|≥ 1 and adjusted *p* -value ≤ 0.05, there were 66 DE lincRNA genes including 50 upregulated and 16 downregulated identified between Day 33 and 65 (Fig. 3A), 29 DE lincRNA genes including 12 upregulated and 17 downregulated identified between Day 65 and 90 (Fig. 3B), 74 DE lincRNA genes including 48 upregulated and 26 downregulated identified between Day 33 and 90 (Fig. 3C). All DE lincRNAs in three groups were distributed in Fig. 3D. In addition, when |fold change|≥ 2 and adjusted *p*-value ≤ 0.01, a total 2638 DE protein-coding genes were identified (Fig. 3E).

### QTL localization and functional enrichment

**Figure 4:**
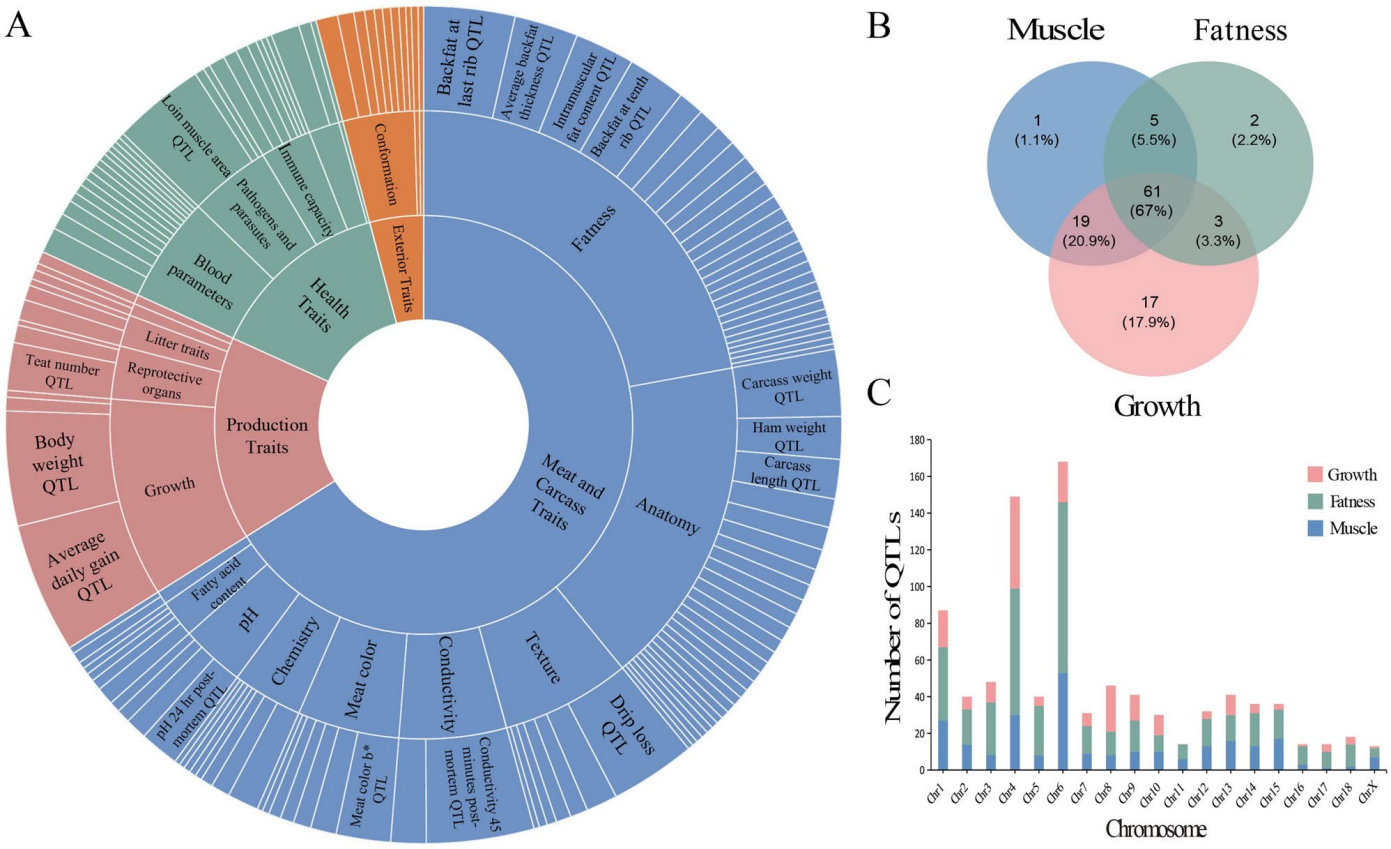
Analysis of the quantitative trait loci of DE lincRNAs. **(A)** The classification and number distribution of QTLs; **(B)** The number distribution of QTLs associated with muscle, obesity, and growth; **(C)** The chromosomal histogram of QTLs associated with muscle, obesity, and growth.

QTL is closely associated with many traits. To explore the relationship between differentially expressed lincRNAs and QTL traits, we performed a correlation analysis by mapping DE lincRNAs to the QTL regions related to pig traits, the pig QTL database contains 31,455 QTLs, representing 695 different traits^24^. Our analysis results showed that 113 of 120 DE lincRNAs were located in 2200 QTL, which corresponded to 331 traits, 27 trait types, 4 trait classes. The greatest number of QTLs were associated with the trait “Meat and Carcass Traits”, accounting for about 59% of the total QTLs. The second highest number of QTL traits “Production Traits” accounted for 11% of the total QTLs (Fig. 4A). We statistically analyzed localization in QTLs associated with muscle, obesity, and growth traits, and found that most DE lincRNAs were targeted at the three trait types. Notably, 100 of 113 DE lincRNAs were closely associated with growth and 86 DE lincRNAs were located in muscle related traits, from this we hypothesized that DE lincRNAs could have an important effect on muscle growth and development (Fig. 4B). Furthermore, we examined the distribution of these QTLs on chromosomes, and found that QTLs were distributed on all chromosomes. Interestingly, the greatest number of QTLs for the three traits were located on chromosome 4 and chromosome 6 (Fig. 4C).

### Prediction of potential target genes of DE lincRNAs

Previous studies have shown that lincRNAs can regulate the expression of target genes by cis or trans via, and participate in the functional regulation of some organisms^25,26^. Firstly, we predicted potential target genes of DE lincRNAs in cis regulation to determine the possible function of DE lincRNAs by searching for protein-coding genes around 100 kb upstream and downstream of DE lincRNAs. We found 303 protein-coding genes were close to DE lincRNAs. GO enrichment analysis showed that 65 of 303 protein-coding genes were assigned to 9 GO terms which mainly involved in the biological processes of transcriptional regulation (Table 2). Furthermore, we performed Pearson correlation analysis revealed that 37 potential target genes (PTGs) were highly correlated with 29 DE lincRNAs (r ≥ 0.8, *p*-value ≤ 0.01). Among them, 12 of 37 PTGs differentially expressed. Meanwhile, most DE lincRNAs were positively correlated with their PTGs. MSTRG.6732 and MSTRG.2061 were significantly correlated with ERGIC1 and HMGB1. Besides, MSTRG.4842 and MSTRG.14169 could regulate their PTGs in two ways: positive regulation and negative regulation. The potential target genes for DE lincRNAs regulation were shown in Table 3.

**Table 2:** GO terms analysis of the nearby protein-coding genes for DE lincRNAs.

GO accession	Term	Count	*p* Value
GO:0,009,952	anterior/posterior pattern specification	8	2.29E−05
GO:0,006,351	transcription, DNA-templated	38	3.46E−04
GO:0,036,444	calcium ion transmembrane import into mitochondrion	3	0.003872665
GO:0,045,944	positive regulation of transcription from RNA polymerase II promoter	20	0.008592517
GO:0,006,376	mRNA splice site selection	3	0.013834427
GO:0,006,355	regulation of transcription, DNA-templated	26	0.017148793
GO:0,009,954	proximal/distal pattern formation	3	0.026743639
GO:0,001,654	eye development	3	0.040439875
GO:0,045,893	positive regulation of transcription, DNA-templated	11	0.049504087

**Table 3:** The correlation between DE lincRNA genes and their adjacent protein-coding genes.

DE lincRNAs	Adjacent protein-coding genes	Pearson correlation coefficient	DE lincRNAs	Adjacent protein-coding genes	Pearson correlation coefficient
MSTRG.98	SYNE1	0.932778133	MSTRG.2696	C17orf105	0.800640629
MSTRG.7542	ZC3HAV1L	0.909721177	MSTRG.243	VGLL2	0.938895472
	KIAA1549	0.802049845	MSTRG.222	7SK	0.923173536
MSTRG.7420	SLC2A4RG	0.878741211	MSTRG.2061	HMGB1	− 0.864645067
MSTRG.7020	PAX1	0.941796265	MSTRG.1905	PIP4K2A	0.820942703
	ENSSSCG00000031878	0.935502944	MSTRG.17805	RAI2	0.992836498
MSTRG.6732	ERGIC1	− 0.886959706	MSTRG.17803	NHS	0.866101456
MSTRG.5732	STAM2	0.89876083	MSTRG.17252	IER5	0.810425662
MSTRG.5387	C10orf71	0.999482502	MSTRG.16842	ssc-mir-125b-1	0.871701204
MSTRG.5199	ACTA1	0.852421469	MSTRG.15750	DLK1	0.909106508
MSTRG.4842	RHOF	0.993619533	MSTRG.14579	CTPS1	0.951838287
	TMEM120B	0.962101405	MSTRG.14169	TUBB6	0.885525513
	WDR66	− 0.898219733		MPPE1	− 0.869796489
	PSMD9	− 0.905954104	MSTRG.13914	MYOM3	0.903239933
MSTRG.4603	COL18A1	0.804671084	MSTRG.12042	HOXC6	0.87413117
MSTRG.4602	COL18A1	0.817579473	MSTRG.11764	PPARA	0.935640688
MSTRG.27	AFDN	0.870481656	MSTRG.11756	ENSSSCG00000035352	0.986041555
MSTRG.2696	MPP2	0.926075239		ENSSSCG00000033576	0.917380642
	MPP3	0.888523664		PPARA	0.84472984
	PPY	0.886803079			

### Functional enrichment analysis of PTGs associated with DE lincRNAs

**Figure 5:**
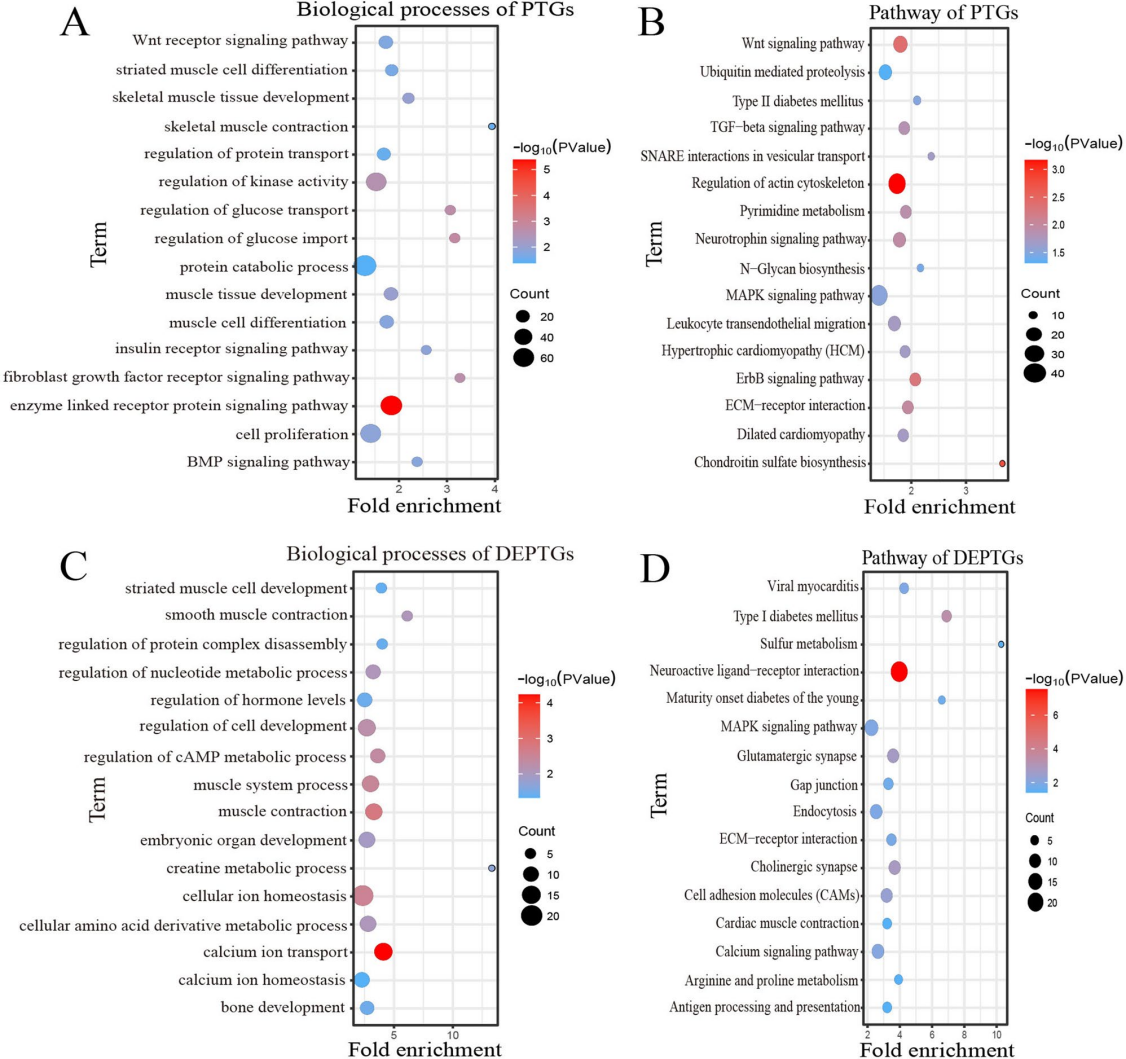
Gene ontology and pathway analysis of PTGs of DE lincRNAs. **(A)** Biological processes analysis associated with muscle growth of PTGs of DE lincRNAs; **(B)** Pathway analysis of associated with muscle growth of PTGs of DE lincRNAs; **(C)** Biological processes analysis associated with muscle growth of DEPTGs of DE lincRNAs; **(D)** Pathway analysis of associated with muscle growth of DEPTGs of DE lincRNAs.

Furthermore, we also predicted the potential target genes from DE lincRNAs in trans regulation, and acquired 4609 PTGs corresponding to 50 DE lincRNAs (r ≥ 0.96, adjusted *p*-value ≤ 0.01). Among these genes, 548 PTGs were differentially expressed in groups as DEPTGs. Which suggested that most of lincRNAs regulated gene expression through trans regulation. GO enrichment analysis showed that 4609 PTGs were enriched in 547 biological processes and 548 DEPTGs were enriched in 287 biological processes. In cases of biological process. Some GO terms were significantly associated with muscle development and energy metabolism, such as skeletal muscle tissue development, muscle contraction, cell proliferation, protein catabolic process, insulin receptor signaling pathway and regulation of glucose transport (Fig. 5A; Fig. 5C). Besides, 4609 PTGs and 548 DEPTGs were enriched in 64 pathways and 28 pathways, respectively. KEGG pathways were involved in Wnt signaling pathway, ECM-receptor interaction, MAPK, calcium signaling, ErbB signaling pathway and TGF-beta signaling pathway (Fig. 5B; Fig. 5D). The results indicated that DE lincRNAs had an important role in regulating their potential target genes regulated composition and growth and development of muscle cells by muscle cells proliferate and differentiate, substance metabolism energy transport and conversion.

### Co expression network analysis of DE lincRNAs and DE potential target genes

**Figure 6:**
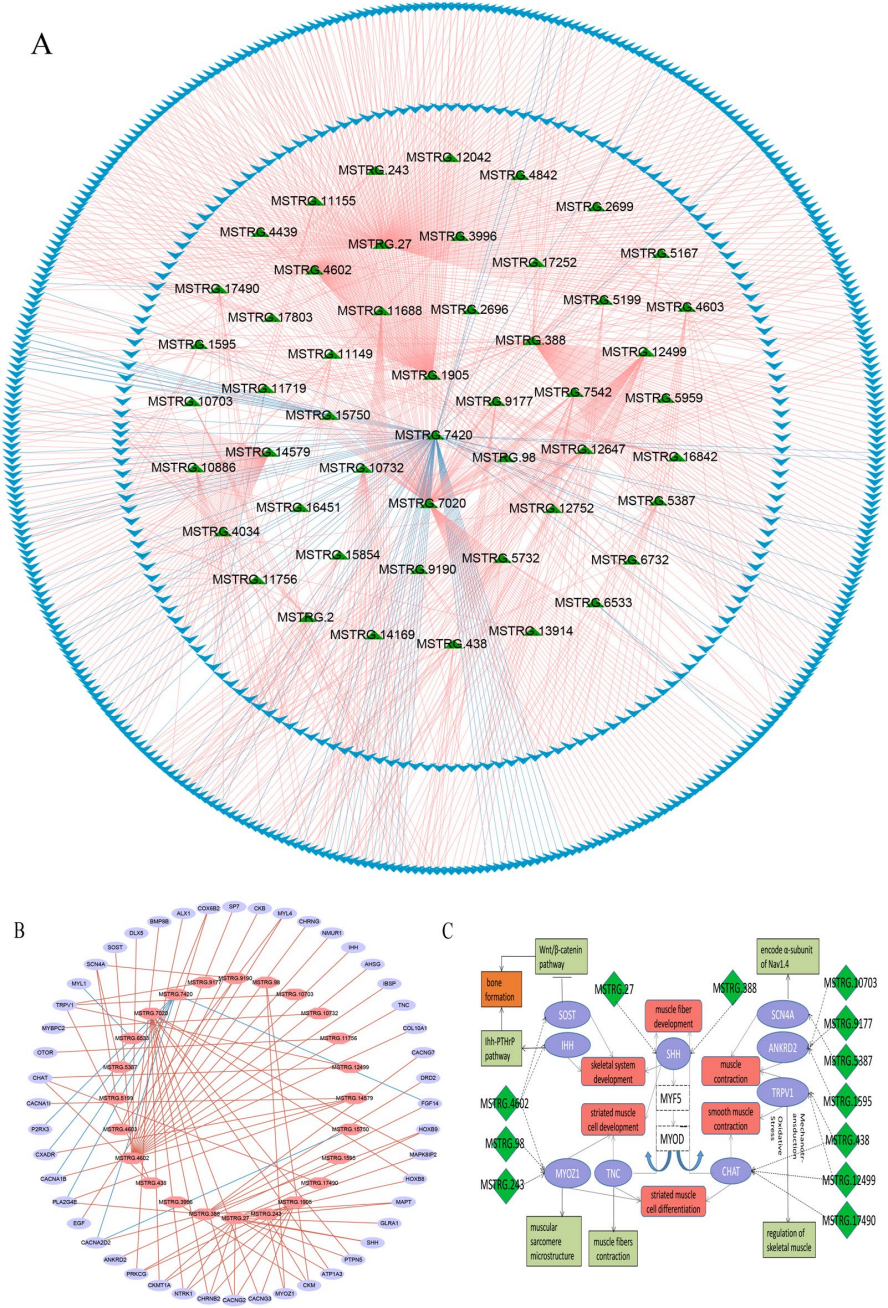
Correlation expression regulation analysis of DE lincRNA genes and their potential target genes. **(A)** Co-expression network diagram between DE lincRNA genes and DEPTGs; **(B)** Co-expression network diagram between DE lincRNA genes and DEPTGs enriched in skeletal muscle development related pathways; **(C)** The interaction of major DE lincRNA genes and DEPTGs enriched in muscle-related pathways.

To understand the relationship of expression between DE lincRNAs and their DEPTGs. The expression regulation relationship between 50 DE lincRNAs and 548 DEPTGs was analyzed, we calculated the interaction of DE lincRNAs and DEPTGs. Pearson correlation analysis results were presented that 860 pairs between DE lincRNAs and DEPTGs with positive correlation and 86 pairs with negative correlation were identified (Fig. 6A). We selected DE lincRNAs and DEPTGs related to skeletal muscle growth and development pathways to construct co-expression networks, and 24 DE lincRNAs exhibited a high co-expression relationship with 48 DEPTGs. Noticeably, DE lincRNA MSTRG.388, MSTRG.4602 and MSTRG.7020 were involved in the regulation of several DEPTGs (Fig. 6B). In order to further explore the function of DE lincRNAs, we investigated nine DEPTGs involving in muscle development related pathways corresponding to 13 DE lincRNAs, we found that SHH targeted by lincRNA MSTRG.27 and MSTRG.388 played an important role in myogenesis (Fig. 6C), and SHH had an essential inductive function in the early activation of the myogenic regulatory factors Myf‐5 and MyoD^27,28^. Besides, lincRNA MSTRG.4602, MSTRG.98 and MSTRG.243 regulated MYOZ1 that encoded calsarcin-2 protein participated in the expression of PPAR-Y2 in skeletal muscle^29^.

### Validation of lincRNA expression levels through qRT-PCR

**Figure 7:**
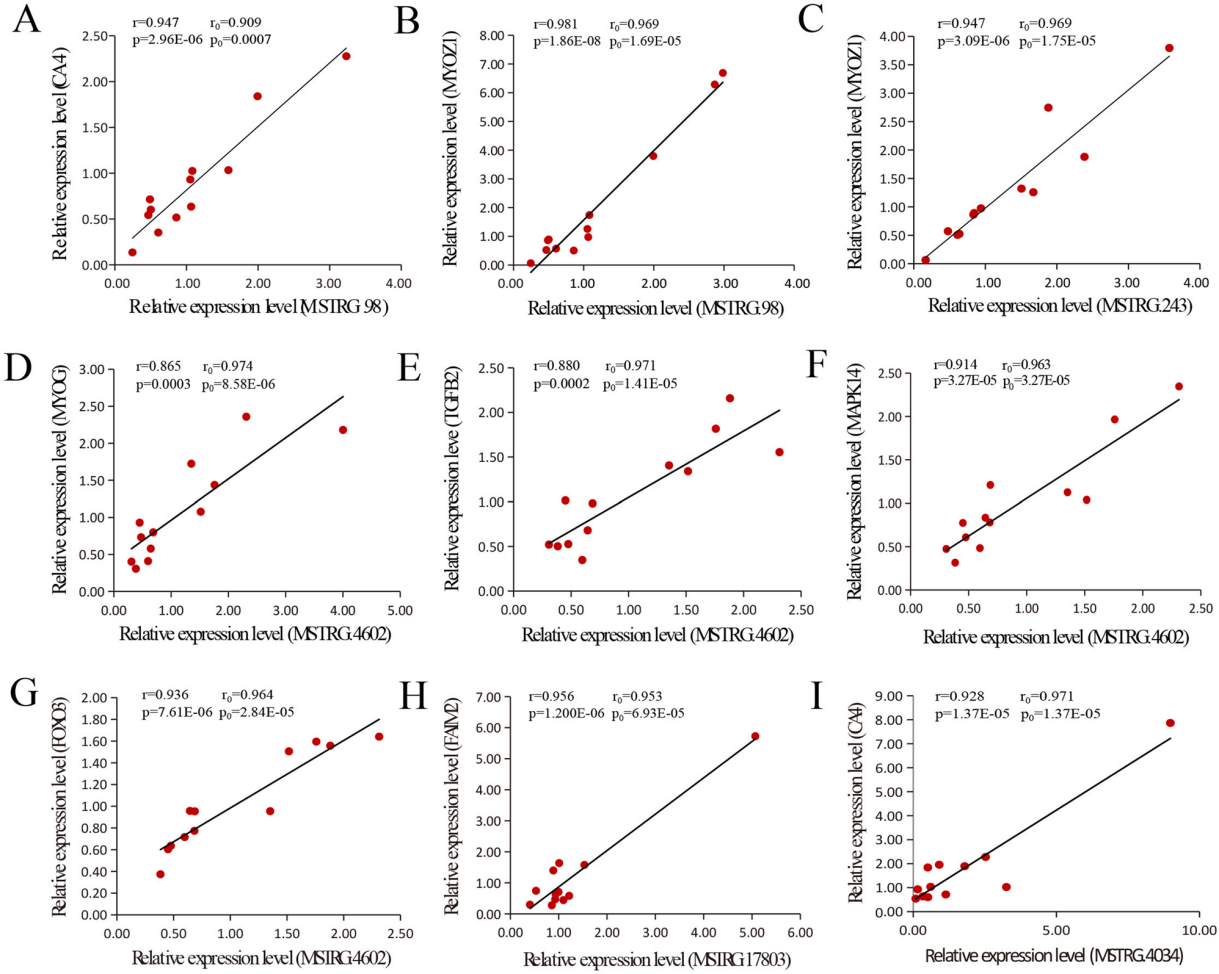
Linear regression of DE lincRNAs and their DEPTGs expression. The r_0_ and p_0_ represent the Pearson correlation coefficient and *p*-value of each pair of differentially expressed lincRNA and its potential target gene, the r and *p* were calculated by qRT-PCR verification experiment. **(A)** MSTRG.98 vs. CA4. **(B)** MSTRG.98 vs. MYOZ1. **(C)** MSTRG.243 vs. MYOZ1. **(D)** MSTRG.4602 vs. MYOG. **(E)** MSTRG.4602 vs. TGFB2. **(F)** MSTRG.4602 vs. MAPK14. **(G)** MSTRG.4602 vs. FOXO3. **(H)** MSTRG.17803 vs. FAIM2. **(I)** MSTRG.4034 vs. CA4.

According to the previous RNA-seq results, we selected nine pairs of DE lincRNA genes and their potential target genes and analyzed their expression levels by qRT-PCR (MSTRG.98 vs. CA4, MSTRG.98 vs. MYOZ1, MSTRG.243 vs. MYOZ1, MSTRG.4602 vs. MYOG, MSTRG.4602 vs. TGFB2, MSTRG.4602 vs. MAPK14, MSTRG.4602 vs. FOXO3, MSTRG.17803 vs. FAIM2, MSTRG.4034 vs. CA4) (Fig. 7). The experimental results showed that the correlation (r^2^) between DE lincRNAs and potential target genes were at above 0.86 and the *p*-values were less than 0.01. The experimental results of the qRT-PCR have a similar tend to the original Pearson correlation coefficient between DE lincRNAs and potential target genes.

## Discussion

Skeletal muscle growth and development are a complex process, which directly determine the meat production and quality in the pig industry. Skeletal muscle is mainly composed of muscle fibers, basement membrane, muscle satellite cells and nerves. Study found that the numbers of muscle fiber have been fixed before the pigs were born, indicating that muscle fiber development is mainly determined during the embryonic period^30,31^. Muscle fiber development takes place in two waves in pig embryonic, the first wave of muscle fiber formation occurs from 30 to 60 days and the second wave occurs from 45 to 90 days^32,33^. In our study, we investigated lincRNAs expression profile in days 33, 65, and 90, which included the primary, second, and final waves of muscle fiber development^33^. Even though the previous studies have showed lincRNAs associated with muscle growth in pig, the dynamic process of expression profile of lincRNAs in embryonic muscle fibers is rare, and our study provides theoretical basis for new exploration in the future.

Based on RNA-seq data published in NCBI, we compared whole gene expression profile in muscle tissue from Duroc in differential development periods. Through a series of transcriptome pipeline analysis, there were 228 putative lincRNAs identified using RNA-seq sequencing, we predicted 37 novel lincRNAs that were not annotated from the nine muscle libraries, which enrich the pig lincRNA annotation and the specific features need to be further investigated in the future. Moreover, we performed a characteristic analysis of putative lincRNAs, involving in transcript and exon length, exon numbers and FPKM, the results showed that the similar characteristic of shorter transcript length, longer exon length, fewer exons, and lower expression levels compared with previous reports^34,35^. Meanwhile, the reliability of the analysis is further improved.

We identified 120 DE lincRNAs and 2638 DE protein-coding genes based on a designed pipeline. Previous studies have shown that there were a large number of lncRNAs located within known QTL regions^36^. To understand the relation between DE lincRNAs and QTLs, we also performed QTL localization analysis for differentially expressed lincRNAs. Some QTLs were involved in large regions, so that multiple genes were located on the same QTL, or multiple QTLs had the same gene location. In among, the specific mechanism may need to be verified by subsequent experiments.

To explore the potential function of DE lincRNAs, we investigated the regulation of lincRNAs on gene expression through cis and trans regulation^26^. For the cis-regulation of DE lincRNAs, we observed that nearby target genes of differentially expressed lincRNAs were related to regulation of transcription, previous studies have confirmed that the porcine lincRNAs were more likely to be enriched in adjacent protein-coding genes that mediate transcriptional regulation, our study was in accordance with Zhao’s report^37^. In addition, some genes have been shown to be associated with muscle cell proliferation and fat deposition. For example, DLK1 was a critical factor in regulating skeletal muscle development and regeneration through Notch dependent^38^. Previous studies found that PPARA was involved in the regulation of fat deposition in porcine subcutaneous fat and longissimus dorsi muscle^39,40^. Besides, myofibrillar structural protein myomesin-3 (MYOM3) was not only associated with muscular dystrophy related proteins and muscle strength, which could be a potential biomarker for monitoring of muscular dystrophy, but was hypermethylated in ischemic cardiomyopathy (ICM)^41,42^. Among them, DEL-MSTRG. 31,882 and its potential target gene with patatin-like phospholipase domain containing 4 (PNPLA4) showed significant positive correlation at the expression level. Therefore, we inferred that DE lincRNAs modulated differences at different stages of development by regulating their potential target genes.

In this study, we identified many target genes of DE lincRNAs that play critical roles in skeletal muscle development. According to these results, we observed that numerous target genes significantly related to DE lincRNAs were involved in the biological processes of skeletal muscle development, such as ACTC1, FOXP1, IGF2BP3, MYOG, MYOZ1, MEF2A, SP1, TGFB2. Previous study reported ACTC1 was implicated in skeletal muscle fiber contraction^43^. In the study by Yang et al., we found that SP1 could act as a central regulator to coordinate skeletal muscle development. IGF2BP3 was a target of SP1 and considered to be a candidate gene for displaying DNA methylation and mRNA expression levels during skeletal muscle development^44^. In addition to the MRFs family, some studies reported MEF2 family could directly regulate myogenesis and morphogenesis^45^. It is noteworthy that SHH targeted by MSTRG.27 and MSTRG.388 is a key transcription factor that regulates the expression of myogenesis and genes related to muscle development. Studies have shown that SHH has significant impact on maintaining MYF5 gene expression and early muscle development^46^.

Subsequently, we investigated Gene Ontology and KEGG pathways analysis of potential target genes of DE lincRNAs, and found that skeletal muscle organ and tissue development processes, muscle contraction, striated muscle cell development were some of significantly enriched GO terms. These results suggest that identified DE lincRNAs have an important impact on the skeletal muscle. Regulation of glucose import, regulation of glucose transport, and insulin receptor signaling pathway also significantly enriched, which were important ways of obtaining and transporting energy, we deduce that DE lincRNAs could participate in the regulatory mechanism of skeletal muscle development through mediating cellular energy responses. Our KEGG pathway analysis showed that significantly enriched pathways including MAPK signaling pathway, TGF-beta signaling pathway, Wnt signaling pathway, ECM − receptor interaction, regulation of kinase activity. Previous studies have confirmed that TGF-beta signaling pathway contributes to muscle development in mice^47^. Moreover, the extracellular matrix (ECM) is a network of structures surrounding muscle fibers, providing a close connection with cell proliferation, differentiation and metabolism. Therefore, we infer that DE lincRNAs could contribute to the differences in skeletal muscle development. In addition, some cardiac diseases, such as viral myocarditis, dilated cardiomyopathy, and hypertrophic cardiomyopathy, were also significantly enriched, these results suggest that DE lincRNAs may have an important effect on myocardial development.

## Conclusion

In the study, we identified 228 putative lincRNAs and analyzed the characteristics of lincRNAs transcriptome compared with protein-coding genes in embryonic muscle tissue of Duroc. We observed numerous differentially expressed lincRNAs and protein-coding genes during differential development stages. Functional enrichment analysis of potential target genes by DE lincRNAs revealed that many lincRNAs participated in muscle growth and development related processes and pathways. Co-expression networks indicated the functional relationship between protein-coding genes and lincRNAs. In summary, our work provides a valuable resource for future research into the potential functions of pig growth and development and is expected to promote the progress of pig production.

## Materials and methods

### Data sources

RNA-seq sequencing data containing nine samples was obtained from the NCBI SRA database. The accession numbers and reads of the RNA-seq data were shown in Table 1. In this study, total male samples were strictly collected from the embryonic muscle tissue of Duroc, and were grouped into three developmental stages (days 33, 65 and 90, three replicates for each stage)^20^. We identified 228 putative lincRNAs and found that many lincRNAs differentially expressed. Moreover, we predicted the potential target genes of DE lincRNAs by cis or trans ways. Gene Ontology and pathways enrichment analysis showed that lincRNAs potentially regulated the processes of protein-coding genes. An interactive network was performed to elucidate the interplay between DE lincRNAs and their potential target genes. This study of skeletal muscle of transcriptome profile will provide a useful resource to further explore the understanding of mechanisms. Besides, elucidating the underlying mechanisms of skeletal muscle growth and development will be helpful for the improvement of production benefits of pig.

### RNA-seq reads mapping and transcriptomic assembly

To ensure the reliability of RNA reads and suitability for the subsequent analysis. FastQC (version 0.11.9) tool (http://www.bioinformatics.babraham.ac.uk/projects/fastqc/) was run to quality control checks on raw sequences data and the sequences of poor quality were trimmed and filtered with Trimmomatic (version 0.36) software to obtain clean reads^48^. The high-quality filtered reads were aligned against the porcine reference genome (Sscrofa11.1 ) using HISAT2 (version 2.0.3) with default parameters^49^. The pig reference genome file was downloaded from Ensembl (ftp://ftp.ensembl.org/pub/release-99/gtf/sus_scrofa/). Next, SAM format files which obtained by mapping were converted to BAM format files with SAMtools (version 0.1.19). After that, StringTie (version 1.3.4) was used to assemble transcripts into nine GTF files, then transcripts of all the samples were combined by the Merge parameter of StringTie into a non-redundant transcript set to produce a uniform transcript GTF^50^. As a result of assembly produced a large amount of novel transcripts, which were mapped to reference annotation file using the GffCompare to discovery novel transcripts information^49^.

### The pipeline lincRNAs identification and analysis

To identify porcine lincRNAs, we performed the following screening of the transcripts obtained after GffCompare, transcripts which the class-code annotated as ‘U’, were more than 200 bp in length and contained at least 2 exons were retained^51^. Next, all remaining transcripts were scored with CPC to determine their coding potential, transcripts of CPC < 0 were considered unable to encode proteins^52^. Then, we translated transcripts sequences into possible protein domains with Transeq^2^ and excluded transcripts that were matched in the Pfam database (E-value < 1e-5)^53^. Furthermore, transcripts that contained similar known proteins in non-redundant reference sequence (NR) database and UniRef90 database were discarded by BLASTX tool (E-value < 1e-5)^54^. Finally, we performed normalization on the transcript by calculating the ‘fragments per kilo-base of exon model per million mapped reads’ (FPKM) using StringTie with the parameter ‘-B’, and transcripts were retained while FPKM was greater than 0.5 in at least a sample^49^.

### Comparison of identified lincRNAs and protein-coding transcripts

At present, the Ensembl database contains comprehensive genetic information for many species. We downloaded the pig reference annotation file that contained 45,788 protein-coding transcripts corresponding to 23,422 protein-coding genes in order to compare the characteristic differences between identified lincRNAs and protein-coding genes. LincRNAs annotation information was downloaded from the ALDB database, we acquired about 12,103 known lincRNA transcripts corresponding to 7,381 lincRNA genes, identified lincRNAs and protein-coding genes were aligned to the corresponding reference annotation files to obtain their detailed information, respectively^55^.

### Differential expression analysis of lincRNAs

We used the python package called ‘HTseq-count’ to calculate the numbers of reads from nine samples^56^, and the resulting count files were used to evaluate the differential expression levels between different groups by the DEseq2 package in R^57^. By screening, lincRNA transcripts with |log2 fold change |≥ 1 and adjusted *p*-value ≤ 0.05 were identified differentially expressed, protein-coding transcripts with |log2 fold change |≥ 2 and adjusted *p*-value ≤ 0.05 were identified differentially expressed.

### QTL location analysis of differentially expressed lincRNAs

To further explore the function of differentially expressed lincRNAs (DE lincRNAs), a correlation analysis was performed between DE lincRNAs with quantitative trait locus (QTL). The pig QTL reference file was downloaded from https://www.animalgenome.org/cgi-bin/QTLdb/SS/index, and the parameter ‘intersectBed’ was used to acquire DE lincRNAs to capture the QTL traits associated with lincRNAs.

### Prediction of potential target genes

We predicted the molecular functions of protein-coding genes regulated by RNA in cis and trans. Firstly, the neighboring protein-coding genes nearby DE lincRNAs (< 100 kb) were identified based on cis-prediction principles using Bedtools. For trans regulation of DE lincRNAs, we calculated the Pearson correlation coefficient (r) between DE lincRNAs and protein coding genes, we selected protein-coding genes that Pearson correlation coefficient ≥ 0.96, adjusted *p*-value ≤ 0.05 as the potential target genes of DE lincRNAs.

### Functional enrichment analysis GO and KEGG

The list of potential target genes was performed to predict biological process and potential signaling pathway based on gene ontology (GO) and Kyoto Encyclopedia of Genes and Genomes (KEGG) in DAVID website (http://david.abcc.ncifcrf.gov/home.jsp)^58,59^. The GO terms and KEGG pathways with *p-*value ≤ 0.05 were considered to be significantly enriched^60–64^. Because of the poor annotation of the pig reference genome, the protein-coding gene IDs were converted into human homologous gene IDs using BioMart from Ensembl.

### Network correlation analysis of DE lincRNA genes and DEPTGs

Network interaction graph can intuitively reflect the relationship between DE lincRNAs and their potential target genes. We select PTGs that Pearson correlation coefficient ≥ 0.96 and adjusted *p*-value ≤ 0.05 differentially expressed in groups were defined as differentially expressed PTGs (DEPTGs), the highly correlated relationship between DE lincRNAs and the underlying potential target genes were established and visualized by Cytoscape (version 3.4).

### Validation of differentially expressed lincRNAs

To verify our analysis results, qRT-PCR was carried out to test the expression between five DE lincRNAs and seven potential target genes which were randomly selected. There were 16 samples from embryonic muscle tissue used for the experiments (each experiment contained three biological replicates). Total RNA was extracted using Trizol reagent according to the manufacturer’s protocols, and reverse transcribed to cDNA using PrimeScript RT reagent Kit with gDNA Eraser r (Takara, Dalian, China). Quantitative PCR was performed using SYBR Premix Ex Taq II on Bio-Rad CFX-96 system (Bio-Rad Laboratories, Hercules). The relative expression levels of all genes were calculated by the 2^–∆∆CT^ method. All primers were designed with Primer 5 program (Table S7).

### Data availability

All the raw data involved in this study could be obtained from public database. This data can be found here: https://www.ncbi.nlm.nih.gov/sra/?term=SRP216286.

### Ethics statement

All the experiments were done in accordance with the relevant guidelines and regulations of animal care and use committee and the study was approved by The Scientific Ethic Committee of Huazhong Agricultural University, Hubei province.

## Supplementary Information

Supplementary Table 1.

Supplementary Table 2.

Supplementary Table 3.

Supplementary Table 4.

Supplementary Table 5.

Supplementary Table 6.

Supplementary Table 7.
